# Hold it there! Dynamic adjustment of fixation duration in visual search tasks

**DOI:** 10.1167/jov.26.6.3

**Published:** 2026-06-04

**Authors:** M. Pilar Aivar, Laura Cepero Amores, Elena Sanz, Miguel A. Vadillo, Victoria Plaza

**Affiliations:** 1 Facultad de Psicología, Universidad Autónoma de Madrid, Madrid, Spain

**Keywords:** visual search, eye movements, fixation durations

## Abstract

Eileen Kowler made very relevant contributions to our understanding of the fine-grain details of oculomotor performance in visual search tasks. She showed that saccadic timing could be strategically adjusted during search, considering aspects like fixation content or available visual information (Wu & Kowler, 2013). Following her steps, in this article we present additional results that support the idea of a dynamic adjustment of fixation durations. In three different experiments we registered eye movements while participants performed different visual search tasks. In two of the experiments only one visual search display was used, which was presented in all trials. In the third experiment half of the displays were repeated over trials. Eye movements in the three tasks showed similar patterns. In all experiments the number of fixations needed to find the target decreased significantly over repetitions. Interestingly, fixation durations differed depending on whether the fixation was on the target item or on other elements. Moreover, display repetition also had an effect on oculomotor performance: the duration of search fixations increased over repetitions while that of target fixations decreased. Amplitudes for search saccades also decreased over repetitions, while fixation locations gradually got closer to the target. These results provide interesting insights regarding oculomotor scanning strategies during search.

## Introduction

Nowadays, we cannot understand vision and attention without considering eye movements. This is so in great part thanks to the work of Eileen Kowler (see Kowler, 2011). We all know that because of the anatomical and physiological properties of our eyes (different kinds of receptors with different sensitivities and a non-homogeneous distribution on the retina, plus complex groupings through receptive fields of different sizes and characteristics) eye movements are vital for visual functioning and are constantly occurring (see Land, 1999). But, as Eileen Kowler showed multiple times during her career, this does not mean that eye movements are generated following a fixed script. On the contrary: “saccadic planning seeks to maximize task performance” (Kowler, 2011, p. 1469). This is the reason why studying eye movements provides very rich information about how we are able to solve tasks: eye tracking data shows *what* we fixate, *when* we fixate it, and *for how long*.

*Where* we look is affected by multiple factors, from low-level aspects of the scene to high-level cognitive aspects, like which specific task is being performed (for a review, see Tatler, Hayhoe, Land, & Ballard, 2011). Overall, the main goal of the saccadic system is to obtain the information that is needed precisely when it is needed (Hayhoe, 2017). In this respect, *when* a fixation occurs is equally important for performance: objects might be completely missed if they are not fixated at the right time (Shinoda, Hayhoe, & Shrivastavah, 2001). Furthermore, fixations can be made in an anticipatory fashion, considering what is going to be needed next (Pelz & Canosa, 2001; Sullivan, Ludwig, Damen, Mayol-Cuevas, & Gilchrist, 2021) and the requirements of the motor system (Matthis, Yates, & Hayhoe, 2018). However, the analysis of other temporal aspects of oculomotor performance, like *how long* we need to hold a fixation to obtain the relevant information, has been largely neglected.

Only a few studies have analyzed which factors influence fixation duration. Initial studies mostly focused on the role of aspects related to oculomotor control. Using very simple tasks they showed that fixation durations might be influenced by previous saccade amplitude (Nattkemper & Prinz, 1987) or reprogramming needs (Abrams, 1992). Some authors even suggested that fixation durations were in part preprogrammed (Vaughan, 1978). When more complex tasks were used the role of perceptual information became more relevant. For example, Prinz, Nattkemper, and Ullman (1992) had participants scan through different lists of random strings of letters to find a predefined target. One of the variables manipulated was target *discriminability*: some of the target letters were easier to detect than others. They found that fixation durations were shorter for the easy targets. These authors also manipulated string complexity and results showed that fixation durations were longer when complexity was high. Other studies have shown that manipulations that increase foveal processing difficulty also produce an increase in fixation duration. This occurs, for example, when the amount of foveal information is reduced with a gaze-contingent moving window: with small window sizes fixation durations increase (Levy-Schoen, O'Regan, Jacobs, & Coëffé, 1984). Degradation of visual information through the use of masks or by a reduction in contrast or luminance also produces similar effects (van Diepen, De Graef, & d'Ydewalle, 1995; Walshe & Nuthmann, 2014).

In the specific context of understanding visual search, initial studies analyzing the timing of saccadic eye movements also showed effects that were mostly linked to target-distractor discriminability. For example, in a task in which participants had to find a complete circle among Landolt Cs with gaps of different sizes, Hooge and Erkelens found that fixation durations increased with increasing difficulty (smaller gap sizes) (Hooge & Erkelens, 1996; Hooge & Erkelens, 1998). A similar result was obtained when line-width was manipulated: when stimuli were easily discriminable fixations were shorter (Hooge & Erkelens, 1999). Similar effects of processing difficulty on fixation duration have been reported using variations of this task, for example when presenting mixed proportions of easy and hard targets (Hooge, Vlaskamp, & Over, 2007) or when using a sequential search (Trukenbrod & Engbert, 2007). Overall, these results suggest that fixation durations are determined to a great extent by the requirements of foveal discrimination. Still, it is not clear whether fixation durations are pre-programmed or are adjusted online depending on the processing needs. For example, after comparing performance in blocked and mixed conditions, Hooge and Erkelens rejected the hypothesis of a direct, fixation-based, control of fixation durations (process-monitoring model) and concluded that fixation durations were mostly pre-programmed, based on the expected difficulty of the task (Hooge & Erkelens, 1996). However, when the proportion of easy and hard targets was manipulated, results were more in agreement with an explanation of fixation durations that considered both components, process-monitoring and pre-programming (Hooge et al., 2007).

Interestingly, multiple studies have also shown that fixation history plays a relevant role in the control of fixation durations. For example, it has been reported that the very first fixation usually has a longer duration (Hooge & Erkelens, 1996; Over, Hooge, Vlaskamp, & Erkelens, 2007; van Loon, Hooge, & van den Berg, 2002; Zingale & Kowler, 1987). Hooge et al. (2007) also found that fixations that occurred immediately after a fixation on a difficult element had longer durations, while the opposite pattern did not occur. In a similar line, a study by Kowler and her team (Wu & Kowler, 2013) found that fixation durations *varied* over the whole sequence of fixations performed during a trial. These authors showed that dwell times depended on several factors, including both the nature of the location fixated and of the next saccadic goal. In their study participants performed a visual search task in a display with *multiple* targets. Two different tasks were compared: (a) finding the targets and estimating the mean tilt of the lines that they included, or (b) merely looking at the targets (with no additional task). In both tasks average fixation durations showed a very clear effect of target discriminability, as in previous work. But Wu and Kowler did not only look at average fixation durations. They also analyzed in detail how other variables, at the trial level, influenced dwell times. The reported results are very interesting: fixations on target items were always *longer* than fixations on non-target elements, and this occurred in both tasks, so it could not be due to response preparation. They interpreted this result as reflecting two different phases during search: exploratory versus targeting.

These authors concluded that “saccadic timing was strategically adjusted as the search proceeded*”* (Wu & Kowler, 2013, p. 17). They highlighted that extending a fixation for too long has a cost, because it increases overall search time, but delaying the next saccade to take advantage of peripheral information might also be beneficial if it improves search efficiency (better targeting means fewer fixations). Therefore, at the oculomotor level participants might use different strategies, which can also change over trials. The subtle variations in fixation duration reflect these strategic adjustments and could also provide information about other aspects of the task that might be used to optimize performance.

Still, there are alternative explanations for these variations in fixation duration at the trial level. For example, Over et al. (2007) analyzed how knowledge about target conspicuity affected saccadic planning in visual search. Participants had to find military objects on natural scenes (varying conspicuity) or a closed square among other squares that included a gap (constant conspicuity). Surprisingly, authors found that in both tasks fixation durations and saccade amplitudes followed a *coarse-to-fine* time course: initial fixations in each trial (excluding the very first one) had shorter durations and higher amplitudes, but progressively fixation durations increased and amplitudes decreased during the trial. This strategy appeared even when it was suboptimal (when conspicuity was known), which contradicts the assumption of a strategic adaptation to the demands of the task. This was interpreted as evidence for an intrinsic coarse-to-fine mechanism in eye movement control.

In our group we are interested in understanding the specific ways in which visual search strategies improve (i.e., Vadillo, Aniento, Hernández-Gutiérrez, Saini, & Aivar, 2024; Vadillo, Giménez-Fernández, Aivar, & Cubillas, 2020). Inspired by Kowler's example of exploring the fine-grain details of visual search, we have systematically analyzed eye movement patterns in the different studies conducted in our lab. This led us to find that results regarding fixation durations were very similar across search tasks of different characteristics. We found this very interesting and relevant in the context of these discussions. For example, we wondered if our data supported the idea of duration adjustments at the trial level or if it fit better with a coarse-to-fine strategy. These are the results that we are reporting in this article. Data comes from three different experiments conducted in our lab. In all experiments participants had to find a unique target among distractors while the search display was repeated over trials. Each study was originally designed with a different goal in mind, but we were surprised to find similar patterns regarding fixation durations. On the one hand, there was an effect of content similar to that reported by Wu and Kowler (2013): search fixations had shorter durations than target fixations. Moreover, display repetition also had a systematic effect on fixation durations over trials. We believe that these results support the idea of a dynamic adjustment of fixation durations.

## Methods

Although the task and stimuli presented varied in each case, all the experiments reported here were conducted using the same equipment. Data analysis was also performed using the same parameters, so all that information is provided here. Specific details regarding each experiment and task are provided in each of the sections below.

### Participants

Students from the School of Psychology of the UAM participated in the different experiments reported here (more details below). All participants had normal or corrected-to-normal vision and declared no problems with color vision. Participants were not excluded if they used glasses or contact lenses and performed the tasks wearing their eye correction. Eye tracker infrared illumination levels were adjusted in each case to obtain good calibration. All participants provided written informed consent prior to participation and received course credit for their time. All the experiments reported here were part of a global study on the characteristics of eye movements in different visual search tasks, which was approved by the UAM Ethics Committee (ref. CEI-94-1724). Sample size for the different experiments was not based on a formal power analysis, but on participants’ availability (all experiments were conducted individually).

### Apparatus

All the experiments were performed in the same lab at different times. Stimulus presentation was carried out using a standard PC computer and eye movements (monocular, left eye) were recorded using an infrared eye-tracker (ASL 6000; Applied Science Laboratories, Bedford, MA, USA), with a frequency of 60 Hz. The spatial error of this system is of less than 1°, according to manufacturers. Stimuli were displayed on a computer monitor (1280 × 1024 LCD), which was placed 74 or 78 cm away from the participant (depending on the experiment). Head position was stabilized with a chin rest. Stimulus presentation and eye tracker operations were controlled with custom-made scripts, programmed in Matlab (The MathWorks, Natick, MA, USA) with the Psychophysics Toolbox extension (Brainard, 1997; Kleiner, Brainard, & Pelli, 2007; Pelli, 1997). The eye tracker was controlled from a second, standard PC computer. Both computers communicated through a parallel port connection.

### Eye data analysis

In all experiments and for all trials we obtained response reaction times and gaze position over time. Raw scan paths were visually inspected first to determine data quality and low-quality recordings showing excessive drift or multiple track losses were eliminated from further analysis (specific data for each experiment is provided below). The amount of data that was removed is in the range of what has been reported in previous studies (for a discussion, see Holmqvist et al., 2011, p. 141).

Eye movement recordings were further processed with Matlab. To detect fixations we used a simple, custom made, velocity based algorithm, similar to the I-VT method described by Salvucci and Goldberg (2000). First, velocity profiles were calculated from the raw data and then a velocity threshold was applied to separate high and low velocity sections. Given the temporal resolution of the eye tracker, no smoothing algorithms were used. Threshold values were adjusted for each experiment, since display items had different sizes. Specifically, we used the following velocity thresholds in each task: Repeated Search with Letters: 1546 pixels per second (approximately corresponds to 30°/sec); Repeated Search with Objects: 1820 pixels per second (about 35°/sec), and Contextual Cueing: 1272 pixels per second (about 25°/sec). Sections above the threshold were considered saccades. Blinks (detected using pupil size data) and missing values were also marked as saccades and removed. The remaining data points were marked as fixations. In a second step, groups of consecutive fixation points with a minimum of *three* data points were collapsed and considered as a fixation. The centroid, or geometrical mean of all the points, was taken as the location for that fixation. When the minimum fixation duration was not met, those data points were classified as saccades. Fixations that occurred outside of the computer screen were eliminated. Fixation durations were calculated by multiplying the total number of data points in a fixation by the inter-sampling period (16.67 msec). Because fixations required a minimum of three data points, the shortest fixations detected lasted a minimum of 50 msec. Such short fixations were not eliminated, since they do seem to occur often in visual search tasks (Velichkovsky, Dornhoefer, Pannasch, & Unema, 2000).

Drift correction was also performed using a custom-made algorithm. The algorithm was based on the idea of *implicitly required fixation locations* (Hornof & Halverson, 2002). It realigned the complete scan path taking as reference those locations that participants had to look at to perform the tasks adequately (fixation point at the beginning of the trial, target item at the end). More details regarding this algorithm can be found in Vadillo et al. (2024).

We determined fixation content by defining a square area around the center of each presented item and calculating how many fixations fell inside it. Area size was also adjusted in each experiment depending on items size (65 pixels for Repeated Search with Letters, 85 pixels for Repeated Search with Objects and 60 pixels for Contextual Cueing). In each trial fixations located in the square area surrounding the target item were defined as *target fixations*, while all the remaining fixations were defined as *search fixations* (excluding initial fixations at the center of the screen). Search fixations included both, fixations on other items in the display and also fixations in empty regions, which occurred quite often (van der Stigchel & Nijboer, 2011; Venini, Remington, Horstmann, & Becker, 2014). For each detected fixation we also determined the amplitude of the preceding saccade in degrees, separating between *search* and *targeting* saccades as done by Wu and Wolfe (2022). Distance to target location (in degrees) was also calculated for each detected fixation.

### Statistical analysis

We used linear mixed-effects models to estimate the general effects of the manipulated variables on performance in each experiment. Our main goal was to obtain an estimation of the effects of the different variables (Meteyard & Davies, 2020), so we added as fixed predictors the different conditions manipulated in each case, together with display repetition, which occurred in all cases. Participants were considered a random factor (with random intercepts). All predictors were included as categorical variables, to obtain specific estimates for each level. No interactions were added to the models to reduce their complexity and to avoid misinterpretation of the results (Brown, 2020). There were also no theoretical reasons to expect interactions between the manipulated variables. Model estimates were obtained with the *lme* function in Matlab, using Restricted Maximum Likelihood as the estimation method. The variance-covariance structure was determined using Cholesky parameterization. Details of each of the models, parameter estimates, and corresponding *t* statistics and *p* values are reported below. No issues appeared regarding convergence of the models.

To analyze whether fixation durations differed depending on their content we used Kruschke's model for a *Bayesian Estimation* of a two-sample *t*-test (Kruschke, 2013). Posterior estimates of the different parameters (group means, standard deviations and differences of means) were carried out using the web implementation developed by Rasmus Bååth (Bååth, 2012), with 20000 burn-in samples. For each variable we report the posterior estimate for each parameter and the 95% Highest Density Interval (HDI). Because we did not have any previous results regarding fixation durations in these tasks we used default non-informative priors, as implemented in the BEST package.

To analyze how repetition modulated fixation durations and saccade amplitudes we calculated simple linear regressions using repetition as predictor. This was done mainly to use slopes to compare repetition effects between the tasks. Regression equations, *t* values and significance levels are provided in each case.

## Repeated search with letters (RSL)

The goal of this study was to analyze whether previous incidental fixations facilitated search when a distractor item was presented as the search target for the first time. We recorded data from two groups of participants in two different experiments. Both experiments only differed in the order in which target items were presented: the critical item appeared as the target in either trial 6 (control) or trial 56. Here we just report the results of one of the two experiments, which served as a control condition (all the targets were presented in each block of trials). The results of the original study were presented at the annual meeting of the VSS (Sanz & Aivar, 2021) and the manuscript is currently under review.

### Participants

Fifteen students between the ages of 17 and 29 years old (19.93 mean age) participated in this experiment (all females).

### Stimuli, procedure and task

**Figure 1.**
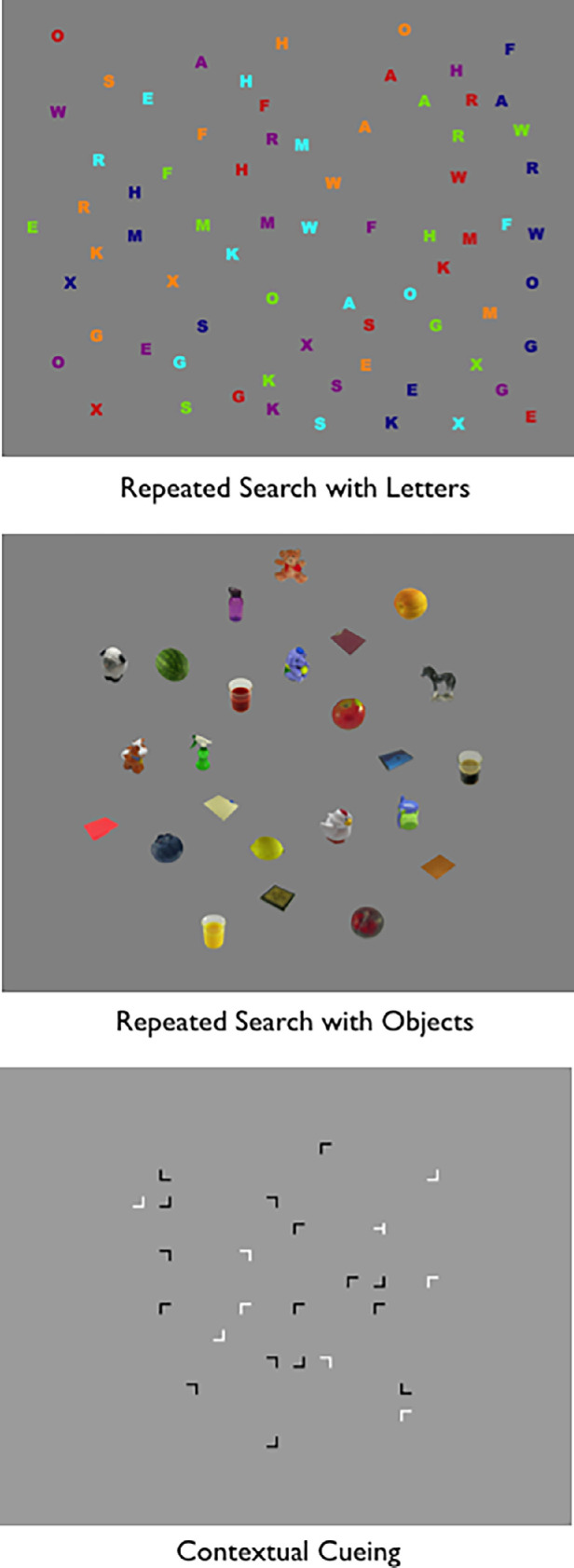
Search displays. The same search display was presented in all trials and for all participants in RSL and RSO. In CC search displays were randomly generated for each participant and half of them were repeated during the experiment.

Participants performed a simple search task: finding the specified target item in the search display. A unique search display was used in *all* trials and for *all* participants (repeated search). The display included 72 letters, presented in font Arial black 36 pt. (see Figure 1). We used all the combinations of 12 different letters (A, E, F, G, H, K, M, O, R, S, W and X) and six colors (red, green, blue, cyan, orange and purple). Letters subtended approximately 1° of visual angle at the viewing distance and were presented on a gray background. To design the search display that was presented in all trials letter locations were chosen with a quasi-random algorithm: random x-y coordinates were obtained for each letter, but the final location was set manually to avoid overlaps and guarantee diversity of colors over the whole display.

Participants searched for the same 12 items (the 12 orange letters), on the same display, a total of six times (72 trials). To allow a direct comparison of eye movements over trials targets were also presented in the same order for all participants. Since the task was easy, no practice trials were presented. A nine-point calibration was performed prior to the start. Once calibrated, participants performed the complete sequence of 72 trials in one continuous session. Each trial began with a one-second white fixation point presented at the center of the screen over a grey background, followed by an image of the target letter, which was also presented at the center of the screen for one second. Then the fixation point reappeared for 500 msec followed by the search display, which remained visible until response or for a maximum of 10 seconds. Participants pressed the space bar when the target was found. Since the task was very easy, participants did not receive feedback on their performance. However, we made sure that they were performing the task as instructed by visually inspecting all the scan paths and determining whether a fixation on the target letter or nearby was found in each trial (see below). Participants were not informed that the search display was the same during the whole experiment. Each recording session lasted between 20 and 30 minutes.

## Repeated search with objects (RSO)

This experiment was a continuation of the previous one, but using images of objects instead of letters. Our aim was again to test whether incidental fixations could facilitate search for a previously irrelevant item. Since incidental retention of visual information is higher for natural objects (Williams, Henderson, & Zacks, 2005) we replicated our first study using pictures. We recorded data from 3 groups of participants in three different experiments. These experiments only differed in the order in which target items were presented. Here we just report the results of the experiment that served as the control condition. The results of the three experiments were presented at VSS (Aivar et al., 2024).

### Participants

Thirty-five students between the ages of 17 and 24 years old (19.47 mean age) participated in this experiment (two males and 33 females).

### Stimuli, procedure and task

This task was similar to the previous one, except that letters were replaced by objects on the search display. 24 different colored natural objects from the Bank of Standardized Stimuli (Brodeur, Dionne-Dostie, Montreuil, & Lepage, 2010) were used to prepare a unique search display (see Figure 1). We wanted a diversity of shapes and colors, so we chose objects from different categories (fruits, toys, office supplies and bottles). Object size was set to 85 by 85 pixels (50 pixels correspond to about 1° of visual angle at the viewing distance). Three concentric invisible circles were used to place the objects, to control for eccentricity effects. Specific locations for each of the objects were chosen using a quasi-random algorithm: random x-y coordinates were obtained for each item, but final location was set manually to avoid overlaps and guarantee diversity of colors over the whole display. Background was gray. As in the previous experiment, the same search display was used in *all* trials and for *all* participants. Targets were also presented in the same order for all participants.

Participants searched for the same 12 items, on the same display, a total of six times (72 trials). No practice trials were presented. Instructions and trial structure was identical to that of the previous experiment. After calibration participants performed the complete sequence of 72 trials in one continuous session. Each recording session lasted between 20 and 30 minutes.

## Contextual cueing (CC)

Details of the original study can be found in Vadillo et al. (2024). In that study we reported the results of 4 different experiments, but eye movements were recorded only in one of them (experiment 4). In this article we provide more details regarding the eye movement measures obtained (not reported on that paper).

### Participants

Twenty-four students participated in the experiment. Average age was 20.46 and about 56% of the participants were female.

### Stimuli, procedure, and task

In each trial participants had to find a rotated T (target) among rotated Ls (distractors). The search display contained a total of 12 or 24 distractors and one target. Items occupied approximately 24 pixels and subtended 0.53° of visual angle. They were placed randomly using an invisible 12 × 12 grid against a gray background. Thirty-two possible target locations (eight per quadrant and equidistant from the center of the screen) were chosen for each participant at the beginning of the experiment. Distractor locations in each display were assigned randomly for each participant. Distractor and target locations never coincided. The target item was always presented in the same color across trials (black or white, randomly chosen for each participant). Displays were composed of relevant and irrelevant items (see an example in Figure 1). Eight distractors had the same color as the target (relevant distractors), while either four or 16 distractors had a different color (irrelevant distractors).

Half of the configurations were repeated along the experiment, intermixed with newly generated configurations. Participants performed 12 blocks of trials. In each block eight old configurations and eight new configurations were presented, in random order, so the complete experiment consisted of a total of 192 trials. In repeated configurations all items (target and distractors) appeared at the same locations, while in new configurations item locations were randomly assigned. Repeated configurations predicted target location, but not correct response, since target orientation was assigned randomly for each participant and trial. Participants were not informed about the repetition of search displays.

A nine-point calibration was performed before the start. Once calibrated participants saw two examples of the task and then completed all the experimental trials. Each trial began with a one-second white fixation point presented at the center of the screen over a grey background, followed by the search display, which remained visible until response. Participants searched for the target item and pressed the letter “z” to indicate that the stem of the T pointed to the left and the letter “m” to indicate that the stem pointed to the right. After an incorrect response a message (“Wrong!”) appeared on the screen for two seconds. Instructions highlighted that the target would always have the same color and that performance could be improved by ignoring all the items presented in the other color. Participants performed all the trials of the experiment in one continuous session, with just one short break after 100 trials. Each recording session lasted between 20 and 30 minutes.

## Results

In all experiments eye movement data from a few participants had to be removed due to poor eye-tracking (presence of noise or artifacts) or because the participant moved during the experiment. This is quite normal when using this methodology (Holmqvist et al., 2011, p. 141). Therefore, regarding eye movement measures we had a total of 12 participants in RSL (80%), 24 participants in RSO (68%) and 20 participants in the CC task (83%). Drift correction was applied, on average, in 22% of trials for RSL, 17% for RSO and 34% for CC. The average drift correction ranged from 0.8° to 1.5°. The root mean square of the inter-sample distances, calculated for each fixation, had average values of 0.14° (RSL), 0.17° (CC), and 0.18° (RSO), which suggest low eye position variability during fixation.

### General performance

Scan paths and eye movement measures showed that participants were performing the tasks as instructed. A fixation on the target letter (as defined above) was found in 98% of the trials in RSL, in 87% of the trials in RSO, and in 83% of the trials in CC. To determine trials in which participants might have responded to the wrong item, we checked whether at least one of the detected fixations was 150 pixels (about 3°) or closer to the target location. In RSL only seven trials (0.8%) did not meet this requirement, although this value was 46 (2%) for RSO. In CC, participants received feedback after each wrong response.

**Figure 2.**
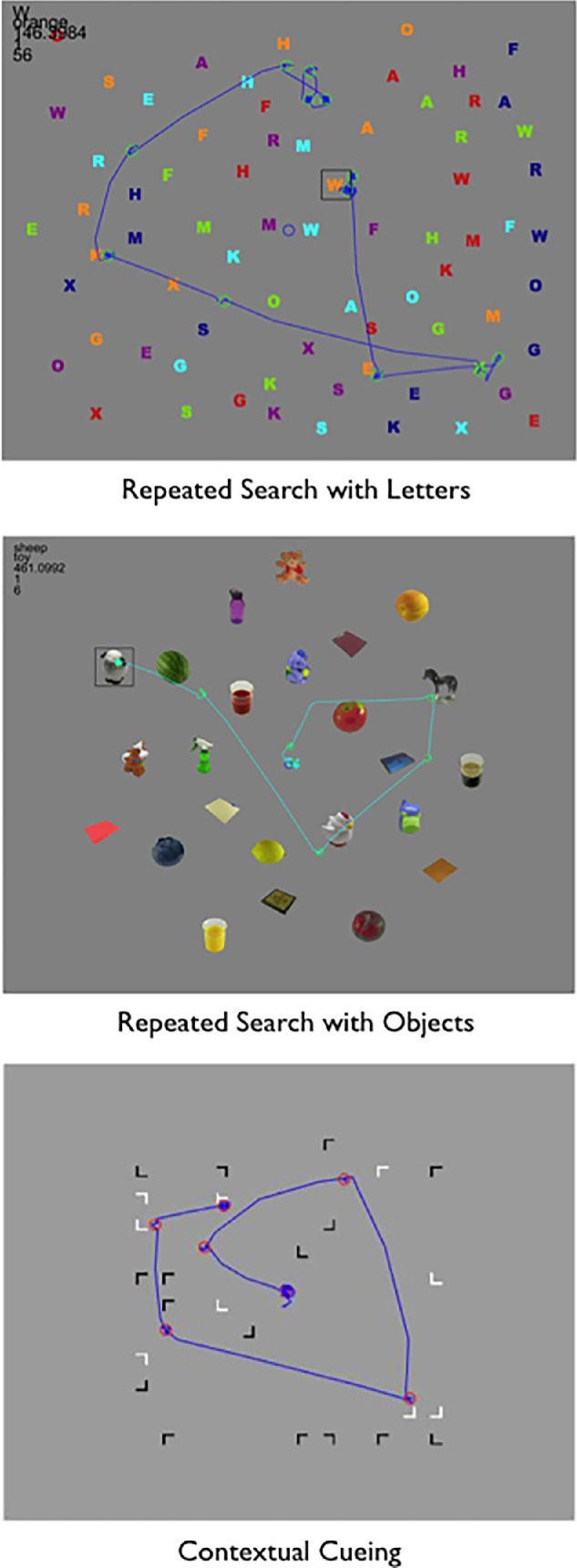
Examples of scan paths in each of the tasks. Lines represent eye trajectory, and small circles represent detected fixation locations. In RSL and RSO the black square indicates target location.

Despite the noteworthy differences between the three search tasks, participants’ eye movements were quite similar, as can be seen in Figure 2. In terms of reaction times the hardest task was RSL, with an overall average reaction time of 1539 msec, followed by CC (1017 msec) and RSO (729 msec). The main factor explaining these differences is the number of items in the display (72 for RSL). The three tasks did all share one common aspect: the repetition of the search display. Therefore we will first provide some results regarding how repetition affected performance.

In all cases we found that target and display repetition led to a gradual reduction in reaction times over trials. LMM were used to obtain estimates of the variations in reaction times due to the effects of repetition and the other variables manipulated in each case. In *RSL* we found that reaction times depended on the specific search target presented [*F*(11, 1063) = 16.172, *p* < 0.0001] but were also modulated by target repetition [*F*(5, 1063) = 6.0939, *p* < 0.0001]. Because target items differed in eccentricity and also local crowding, these differences between items were not surprising. Overall, there was an average decrease of 390 msec between the first and the sixth search according to the model (see estimates in Table 1). This model explained 17% of the variance (adjusted *R*^2^). In *RSO* we found that reaction times also depended on the specific search target presented [*F*(11, 2431) = 15.3, *p* < 0.0001]. These effects were expected, because target items differed in eccentricity. There was also a clear effect of target repetition [*F*(5, 2431) = 89.4, *p* < 0.0001]. The average decrease between the first and the sixth search was also estimated in more than 400 msec (see estimates in Table 2). This model explained 28% of the variance (adjusted *R*^2^). In *CC* reaction times also varied depending on condition [*F*(3, 4593) = 11.568, *p* < 0.0001] and block [*F*(11, 4593) = 44.637, *p* < 0.0001]. There were four different conditions in this experiment, depending on whether the display was old or new and the number of irrelevant distractors (four or 16), and we found that reaction times were slowest when the display included more irrelevant distractors (a longer discussion of these results can be found in Vadillo et al., 2024). Regarding display repetition (blocks), the model estimated an average decrease of more than 480 msec between the first and the last search (see estimates in Table 3). This model explained 38% of the variance (adjusted *R*^2^).

**Table 1.** Repeated search with letters: estimated coefficients, standard errors, *t*-values and significance for the mixed-model fitted to the reaction Times (*1* *+* *Target* *+* *Repetition* *+* *(1|Participant)* in the Wilkinson-Rogers notation). Since Matlab uses the lowest numbered category as the reference group, the first presentation of target 1 (letter W, lowest eccentricity) was used as reference.

Parameter	Estimate	*SE*	*t* value	Sig.
Intercept	1319.9	108.63	12.15	0.000
Target = 2	249.1	117.35	2.1226	0.034
Target = 3	32.489	117.35	0.27684	0.781
Target = 4	352.58	117.35	3.0044	0.002
Target = 5	1148.2	117.35	9.7844	0.000
Target = 6	789.46	117.35	6.7271	0.000
Target = 7	554.69	117.35	4.7266	0.000
Target = 8	215.72	117.35	1.8382	0.066
Target = 9	708.2	117.35	6.0347	0.000
Target = 10	561.01	117.35	4.7805	0.000
Target = 11	581.73	117.35	4.957	0.000
Target = 12	266.62	117.35	2.2719	0.03
Repetition = 2	−293.33	82.982	−3.5348	0.000
Repetition = 3	−212.77	82.982	−2.5641	0.010
Repetition = 4	−181.54	82.982	−2.1877	0.028
Repetition = 5	−372.55	82.982	−4.4895	0.000
Repetition = 6	−390.85	82.982	−4.71	0.000
Random effects:				
Participant	175.12			
Residual	787.24			

**Table 2.** Repeated search with objects: estimated coefficients, standard errors, t-values and significance for the mixed-model fitted to the Reaction Times (*1 + Target + Repetition +(1|Participant)* in the Wilkinson-Rogers notation). Because Matlab uses the lowest numbered category as the reference group, the first presentation of target 1 (the apple, lowest eccentricity) was used as reference.

Parameter	Estimate	*SE*	*t* value	Sig.
Intercept	973.91	38.607	25.226	0.000
Target = 2	45.75	35.503	1.288	0.197
Target = 3	30.397	35.503	0.856	0.391
Target = 4	−27.142	35.503	−0.764	0.444
Target = 5	−10.417	35.503	−0.293	0.769
Target = 6	55.289	35.503	1.557	0.119
Target = 7	28.765	35.503	0.810	0.417
Target = 8	159.78	35.503	4.500	0.000
Target = 9	21.946	35.503	0.618	0.536
Target = 10	235.86	35.503	6.643	0.000
Target = 11	83.892	35.503	2.363	0.018
Target = 12	278.44	35.503	7.842	0.000
Repetition = 2	−274.80	25.104	−10.946	0.000
Repetition = 3	−359.44	25.104	−14.318	0.000
Repetition = 4	−422.49	25.104	−16.829	0.000
Repetition = 5	−442.05	25.104	−17.609	0.000
Repetition = 6	−417.26	25.104	−16.621	0.000
Random effects:				
Participant	142.55			
Residual	358.56			

**Table 3.** Contextual cueing: estimated coefficients, standard errors, *t*-values and significance for the mixed-model fitted to the Reaction Times (*1 + Condition + Repetition + (1|Participant)* in the Wilkinson-Rogers notation). because Matlab uses the lowest numbered category as the reference group, the first condition (new display and 4 irrelevant distractors) was used as reference (Condition 2 was new display and 16 irrelevant distractors; Condition 3 was old display and four irrelevant distractors, and Condition 4 was old display and 16 irrelevant distractors).

Parameter	Estimate	*SE*	*t* value	Sig.
Intercept	1435.7	61.673	23.28	0.000
Condition 2	58.795	16.083	3.6556	0.000
Condition 3	−30.976	16.083	−1.9259	0.054
Condition 4	30.251	16.083	1.8809	0.060
Repetition = 2	−328.96	27.857	−11.809	0.000
Repetition = 3	−365.97	27.857	−13.137	0.000
Repetition = 4	−426.21	27.857	−15.3	0.000
Repetition = 5	−422.69	27.857	−15.173	0.000
Repetition = 6	−444.33	27.857	−15.95	0.000
Repetition = 7	−448.97	27.857	−16.117	0.000
Repetition = 8	−471.78	27.857	−16.936	0.000
Repetition = 9	−437.58	27.857	−15.708	0.000
Repetition = 10	−451.3	27.857	−16.2	0.000
Repetition = 11	−447.59	27.857	−16.067	0.000
Repetition = 12	−487.53	27.857	−17.501	0.000
Random effects:				
Participant	282.21			
Residual	386			

There were also differences between the three tasks regarding the number of fixations needed to find the target: on average participants needed 7.5 fixations in RSL, about 3.7 fixations in CC and about 3.5 fixations in RSO (ignoring the initial fixation at the fixation point). It is not surprising that RSO and CC showed very similar patterns regarding fixations: there were always 24 items on the display in RSO, and either 13 or 25 items in CC. To analyze the effect of repetition we applied the same LM Models used above to the number of fixations and obtained similar effects in all cases. Regarding display repetition effects, and when comparing the first and the last search in each case, the models estimated a decrease of about 2 fixations for RSL [*F*(5, 847) = 4.3133, *p* < 0.0001], a decrease of about 1 fixation for RSO [*F*(5, 1711) = 15.107, *p* < 0.0001], and a decrease of about 1.5 fixations for CC [*F*(11, 3825) = 15.676, *p* < 0.0001].

**Figure 3.**
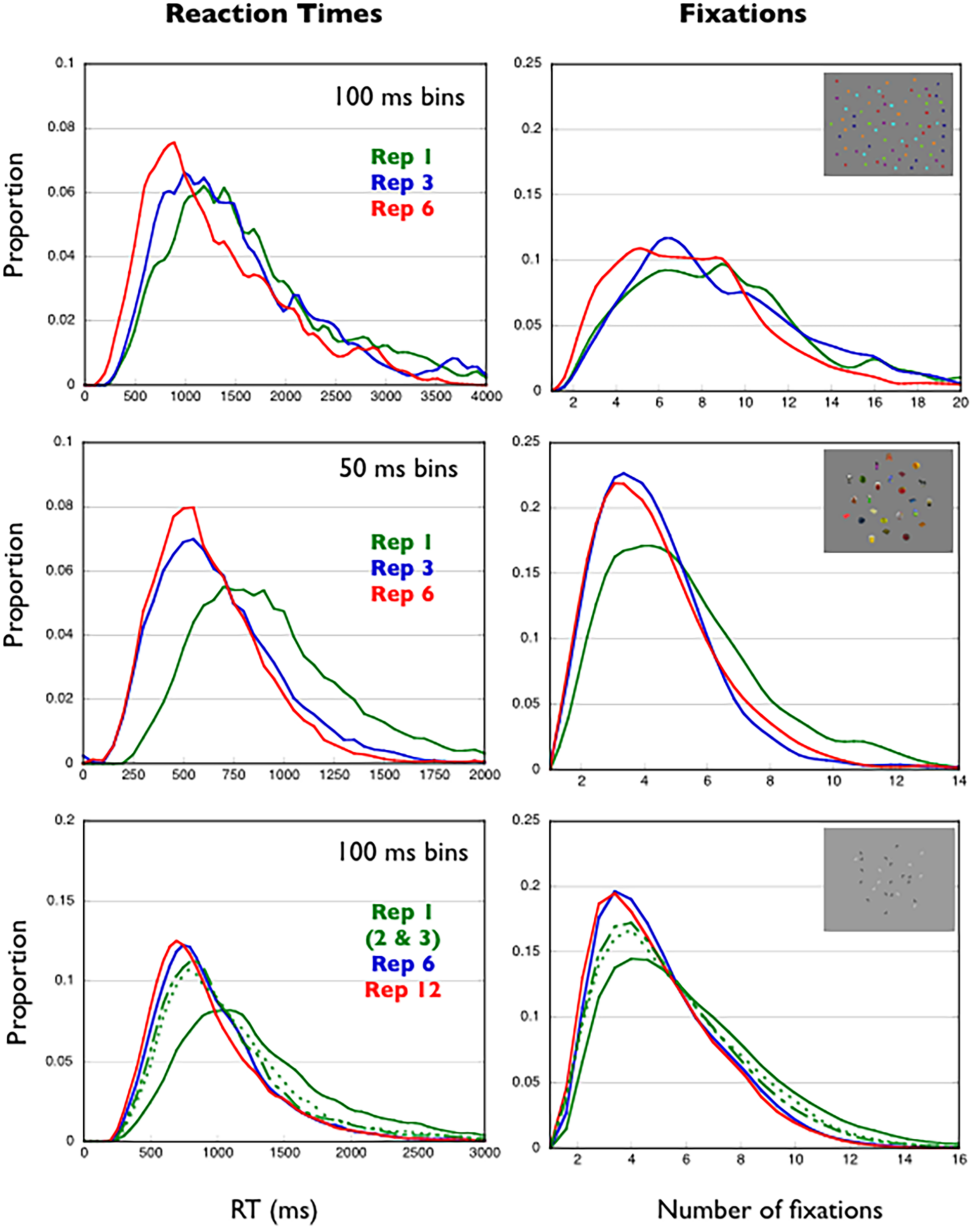
Effects of repetition in the three experiments (from top to bottom: RSL, RSO, and CC). Plots show smoothed histograms with the distribution of reaction times (left) and number of fixations (right), calculated over all participants and trials. Data is grouped by repetition (first search episode in green, last search episode in red).

To illustrate more graphically the effects of repetition in the three experiments Figure 3 shows smoothed histograms with the distributions of reaction times (left) and number of fixations (right), over all participants and experimental conditions, depending on repetition (first search block in green, last search block in red). In all cases target and display repetition produces a shift of the distributions, leading to shorter reaction times and fewer fixations needed to solve the task.

### Fixation durations

**Figure 4.**
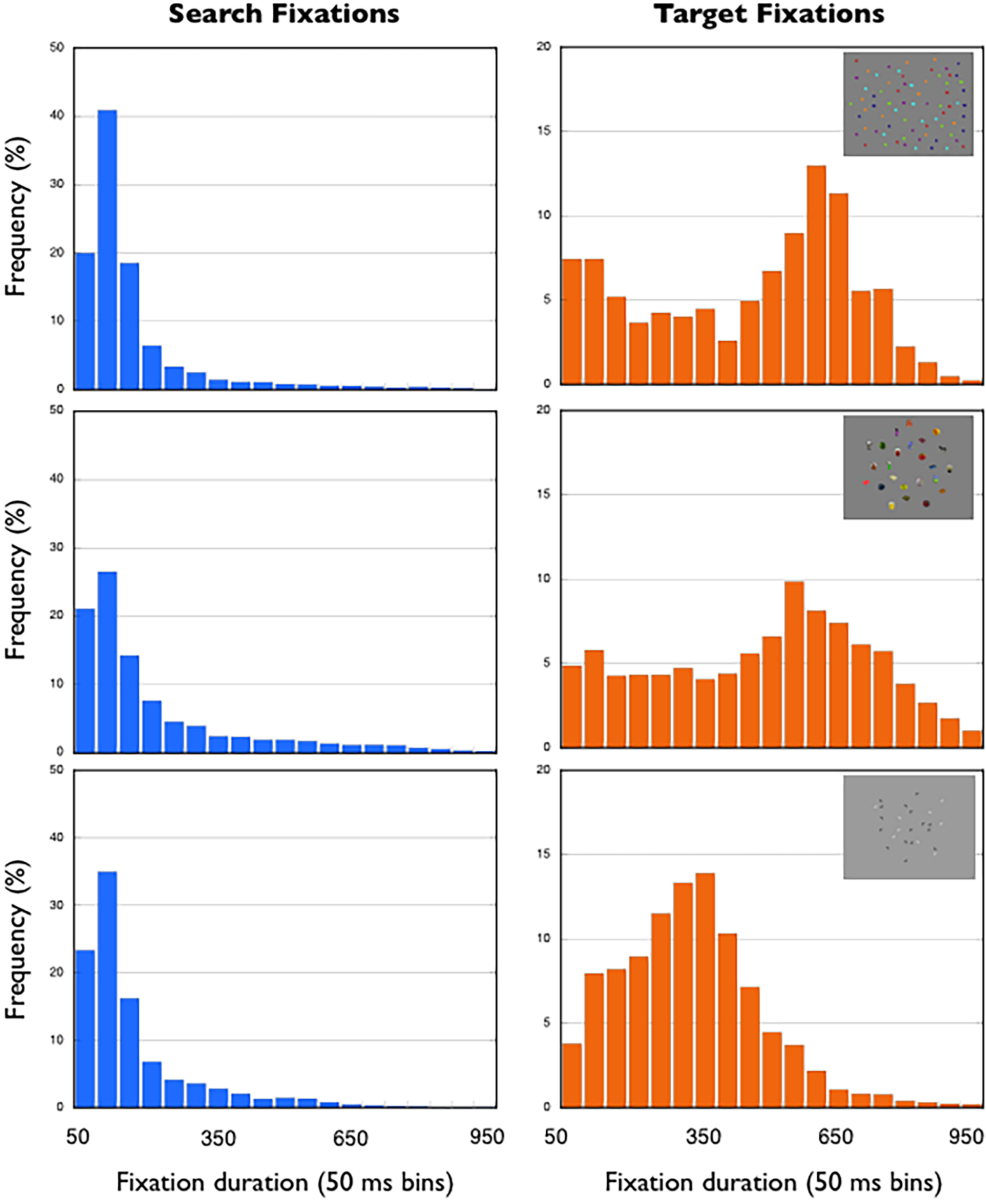
Distribution of fixation durations. Fixations are divided in search fixations (blue) and target fixations (orange). Histograms represent the percentage of fixations in each bin over all trials and participants. Bin size is 50 msec, starting with a minimum duration of 50 msec. Each row represents the results in each of the three tasks (RSL, RSO, and CC).

As explained above, fixations were divided into two groups depending on content: Search fixations and Target fixations. Figure 4 shows histograms with the relative distribution of both kinds of fixations according to their durations for the three tasks (data from all participants and trials taken together). In all cases the same pattern emerges: Search fixations (in blue) mostly show short durations, whereas Target fixations (in orange) have usually much longer durations. In the three experiments we see that 45% or more of the search fixations last up to 150 msec and that about 70% of search fixations or more last less than 200 msec. On the other hand, the distribution of target fixation durations is much more spread in all cases, with the highest proportion of fixations showing a duration over 200 msec.

To compare these two distributions, we obtained Bayesian estimates of the differences in duration between Search and Target fixations using the BEST method (more details of the Bayesian estimates can be found in Table 4). For *RSL* we found that the estimated difference of means was −412 msec [95% HDI: −430 to −393], with a posterior effect size (Cohen's *d*) of −3.32 [95% HDI: −3.36 to −2.97]. Since the HDI for the difference does not include 0, we can conclude that there is a credible difference between the two groups: target fixations clearly had longer durations. Posterior standard deviations also differed between both groups (mean difference of –135 msec [95% HDI: −148 to −120]). A similar pattern was found for *RSO*. The posterior estimated difference of means between both groups also had a value of −412 msec [95% HDI: −426 to −397] and the posterior standard deviations differed between both groups (mean difference of −109 msec [95% HDI: −121 to −97.7]). The posterior mean of the standardized effect size in this case was −3.12 [95% HDI: −3.35 to −2.89]. For *CC* the posterior estimated difference of means between both groups had a value of –202 msec [95% HDI: –207 to −196] and the posterior standard deviations also differed (mean difference of –59.4, [95% HDI: –63.8 to −55.2]). The posterior effect size had a value of –2.45 [95% HDI: –2.57 to –2.35]. Although average differences varied between tasks, all estimates and the large effect sizes suggest that there is a credible difference in duration between Search and Target fixations.

**Table 4.** Posterior estimates of the relevant parameters obtained using the BEST method, for each of the tasks. The 95% HDI is reported between brackets next to each estimate.

	Search fixations	Mean: Target fixations	SD: Search fixations	SD: Target fixations	Normality parameter	Effect size
Repeated search letters	121 [119, 122]	532 [514, 550]	37 [35.6, 38.4]	172 [157, 185]	0.151 [0.130, 0.173]	−3.32 [−3.66, −2.97]
Repeated search objects	131 [128, 134]	543 [528, 556]	66 [62.7, 69.6]	175 [163, 186]	0.0645 [0.043, 0.087]	−3.12 [−3.35, −2.89]
Contextual cueing	121 [120, 123]	323 [318, 328]	46.9 [45.6, 48.4]	106 [102, 111]	0.187 [0.171, 0.203]	−2.45 [−2.57, −2.35]

### Saccade length versus fixation durations

**Figure 5.**
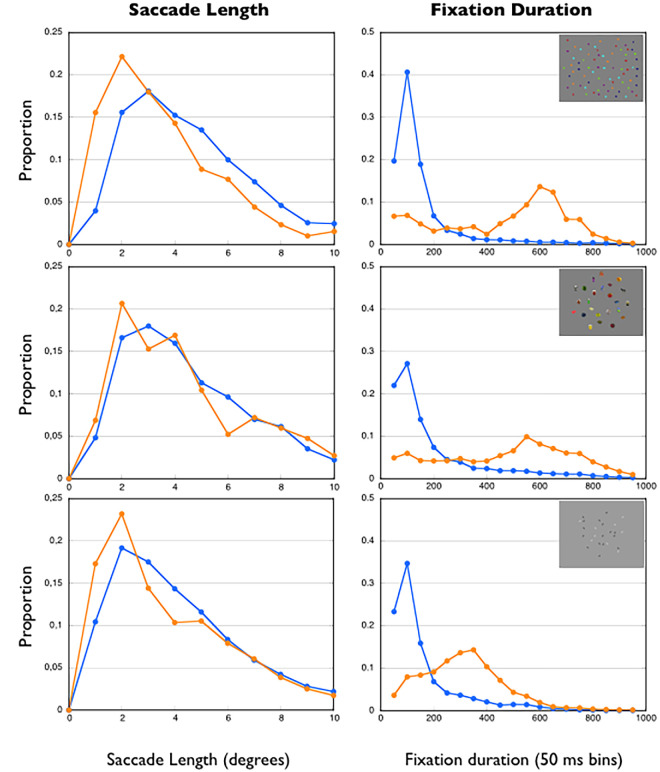
Proportion of saccades as a function of saccade length (left) and fixation duration (right). Lines represents average functions for Search saccades (blue) and Target saccades (orange), calculated as in Wu and Wolfe (2022). Each row represents the results in each of the three tasks (RSL, RSO, and CC).

Once fixations were categorized as either search or target fixations it was also possible to analyze whether there were differences in the amplitude of the saccade preceding each fixation. This resulted in two groups of saccades: search versus targeting saccades, as in Wu and Wolfe (2022). Figure 5 shows saccade length (left) and fixation duration (right) histograms for both groups of saccades. For these plots we first obtained individual distributions and then calculated global averages, using 1° bins for saccade length and 50 msec bins for fixation durations.

Regarding saccade length our results are very similar to those reported by Wu and Wolfe (2022): in the three tasks average saccade lengths were slightly shorter for targeting saccades (*RSL*: 3.35° *RSO*: 4.08° *CC*: 3.35°) than for search saccades (*RSL*: 4.47°; *RSO*: 4.23°; *CC*: 3.78°). However, these differences only reached significance for two of the three tasks (*RSL*: *t*(20.66) = 3.758, *p* = 0.0012; *CC*: *t*(32.21) = 3.4734, *p* = 0.0015). In RSO we see that the distributions of search and targeting saccades practically overlap (*RSO: t*(45.95) = 0.89, *p* = 0.3745). This could reflect better saccadic accuracy in the case of RSO. In this task items had bigger sizes, were clearly different from each other and suffered less crowding. Therefore saccades might have aimed at specific objects, instead of display areas. This strategy would result in less variation in saccade lengths, independently of whether the fixated element was a target or a distractor. For RSL and CC shorter lengths in the case of targeting saccades might reflect the use of peripheral information to determine target location during the previous fixation.

Just for comparison we also analyzed fixation durations using the same methods. In this case the differences between search and target fixations were very clear. Target fixations had longer average durations (*RSL*: 487.70 msec; *RSO*: 527.92 msec; *CC*: 336.12 msec) than search fixations (*RSL*: 168.35 msec; *RSO*: 265.29 msec; *CC*: 176.24 msec), and these differences were significant [*RSL*: *t*(11.51) = −9.5642, *p* < 0.0001; *RSO*: *t*(44.08) = −8.4497, *p* < 0.0001; *CC: t*(21.12) = −7.9212, *p* < 0.0001].

### Effect of repetition

**Figure 6.**
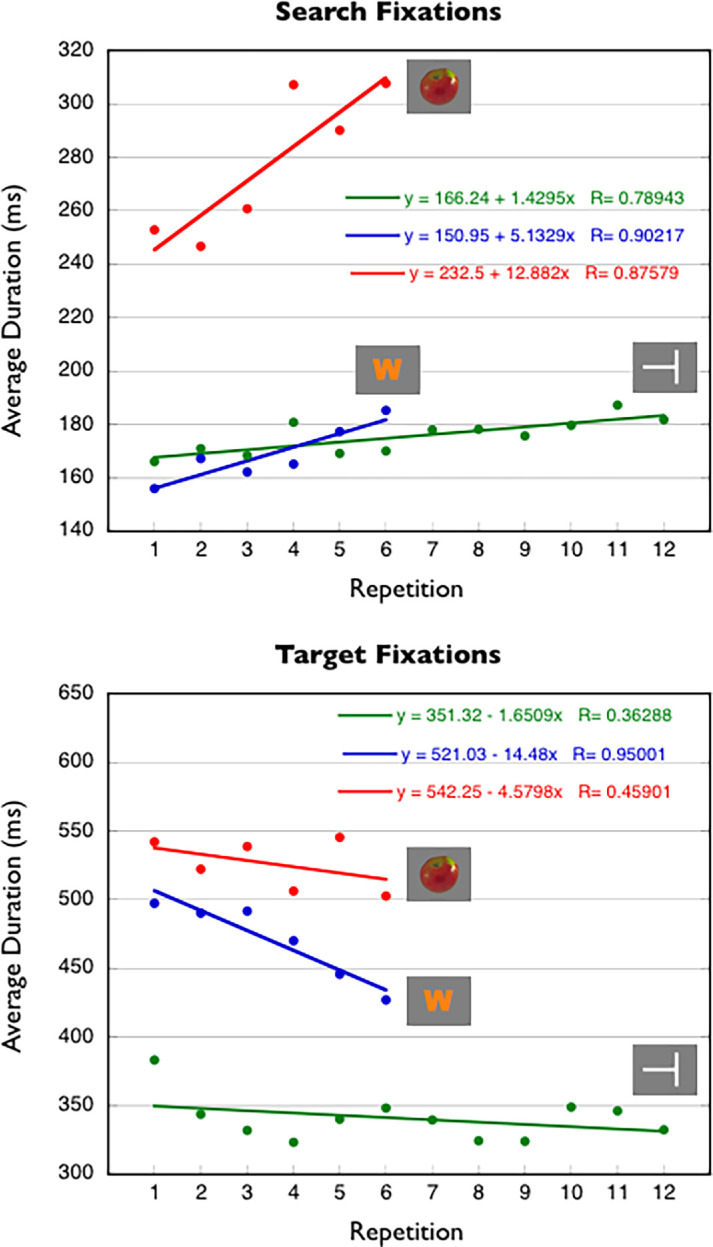
Effect of repetition on fixation duration. Fixations are divided into Search fixations (top) and Target fixations (bottom). Each line represents the linear regression obtained using repetition as a predictor. Each task is represented by a different color (RSL in blue, RSO in red, and CC in green).

As explained above, in RSL and RSO participants searched for all the target items a total of six times, whereas in CC they performed 12 blocks of trials in which half of the displays were repeated. Given that in all the tasks targets and displays were repeated over trials we also analyzed whether fixation durations varied because of repetition. This was interesting, because a very similar pattern of results emerged for the three tasks. These results are plotted in Figure 6, as a linear regression. Linear regressions were mainly used because they allow us to use slopes as an easy way to compare the three different tasks. Search Fixations (top) showed a systematic *increase* in duration over repetitions, although its slope varied for each task (*RSL* in blue, *RSO* in red, *CC* in green). But in all cases, there was a significant positive relationship between both variables (*RSL: t*(5631) = 4.6732, *p* < 0.0001; *RSO: t*(4370) = 4.4779, *p* < 0.0001; *CC: t*(10978) = 3.8831, *p* = 0.0001). This indicates that fixations had longer durations after several repetitions of target and display. Target Fixations (bottom), on the other hand, showed a systematic *decrease* in duration over repetitions, which was very accentuated for RSL [*t*(846) = −2.9589, *p* < 0.0032], barely significant for CC [*t*(3216) = −1.9896, *p* = 0.0467], and did not reach significance for RSO [*t*(1501) = −1.0465, *p* = 0.2954]. This suggests that visual strategies change over time, and that such change affects each phase (search vs. targeting) differently.

**Figure 7.**
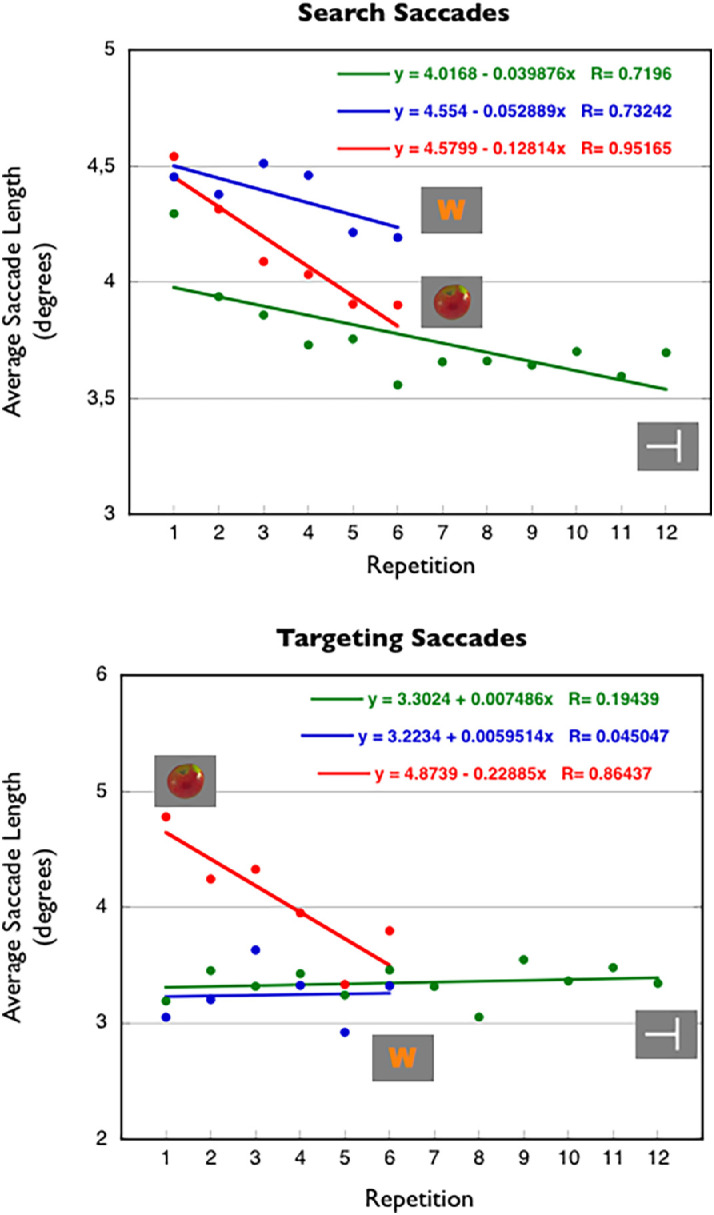
Effect of repetition on saccade length. Saccades are divided into Search saccades (top) and Targeting saccades (bottom). Each line represents the linear regression obtained using repetition as a predictor. Each task is represented by a different color (RSL in blue, RSO in red, and CC in green).

**Figure 8.**
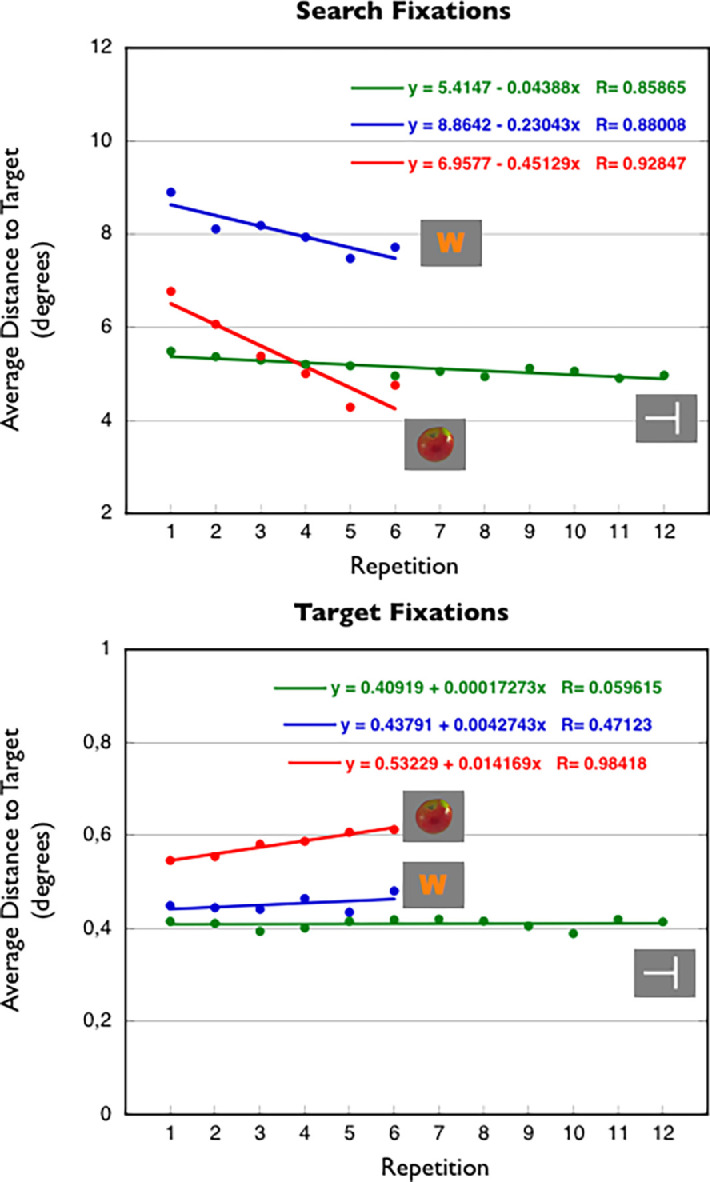
Effect of repetition on distance to target location. Fixations are divided into Search fixations (top) and Target fixations (bottom). Each line represents the linear regression obtained using repetition as a predictor. Each task is represented by a different color (RSL in blue, RSO in red, and CC in green).

Seeing these trends we also analyzed whether target and display repetition had an effect on saccade length. Figure 7 shows average lengths for search and targeting saccades over repetitions for each task, including linear regressions. For search saccades we found a systematic *decrease* in saccade length over repetitions in the three tasks [*RSL*: *t*(5625) = −2.2006, *p* = 0.0278; *RSO*: *t*(4319) = −5.6446, *p* < .0001; *CC*: *t*(10973) = −6.1827, *p* < 0.0001]. For targeting saccades, however, the effect of repetition on saccade length only appeared in one of the tasks [*RSO*: *t*(1498) = −5.5358, *p* < .0001]. Although indirectly, we think that these results suggest an improvement in saccadic aiming over time: overall fewer fixations are made, but to more optimal locations. If this were the case, we would also expect fixations to get closer to the target location over repetitions, which is what we find. We determined the distance from each fixation location to the center of the target, for both search and target fixations (Figure 8). The statistical analysis showed a systematic *decrease* in the distance to the target over repetitions for search fixations in the three tasks [*RSL*: *t*(5626) = −6.5357, *p* < 0.0001; *RSO*: *t*(4332) = −15.987, *p* < 0.0001; *CC*: *t*(10975) = −5.3072, *p* < 0.0001] and almost no variations for target fixations. Only RSO showed a significant effect, with fixation locations getting slightly *further away* from the center of the target over repetitions [*t*(1501) = 3.7809, *p* = 0.0001]. We will discuss the meaning of this result later.

### Order effects

As explained above, we found that *search fixation durations* varied over repetitions. To try to explore this effect in more detail and avoid possible misinterpretations we also analyzed these changes considering the effect of fixation order. For example, a coarse-to-fine pattern in fixation durations and saccade amplitudes might produce changes that seem to reflect learning (Over et al., 2007). With this additional analysis we try to clarify if the changes reported above can be linked to the repetition of the displays or if they can be explained in other ways.

**Figure 9.**
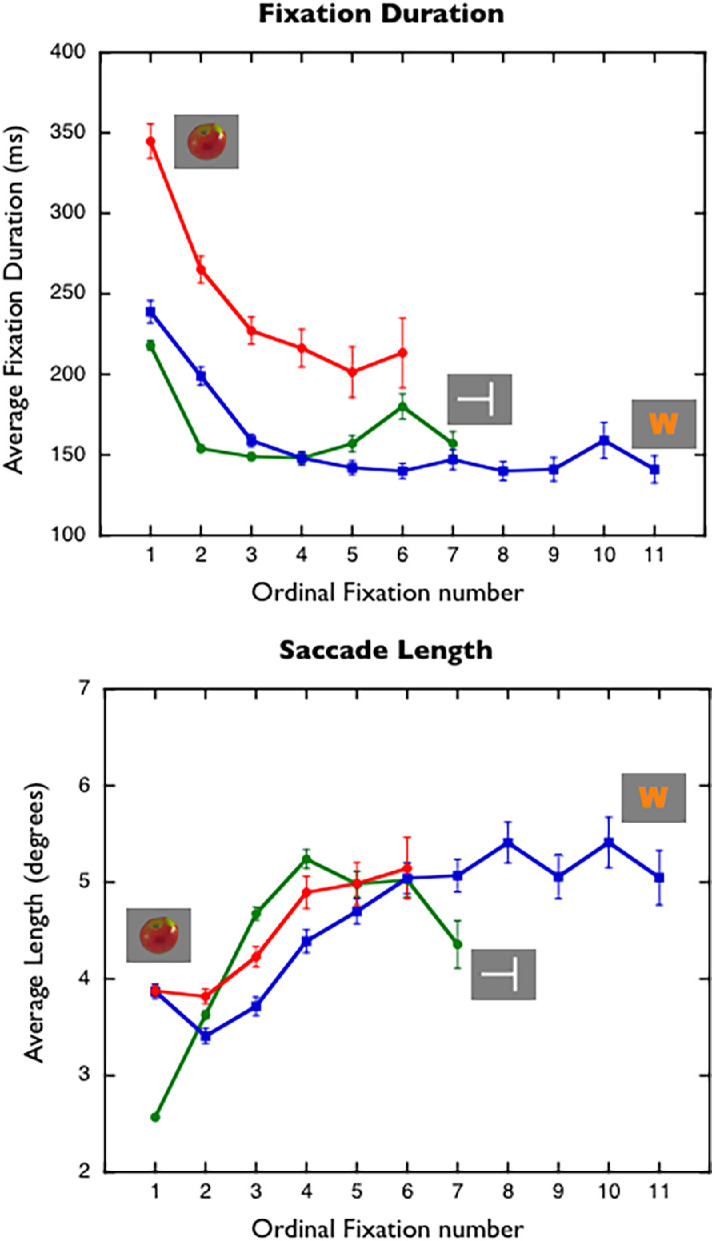
Mean fixation duration (top) and saccade length (bottom) as a function of ordinal fixation number (only search fixations). Each task is represented by a different color (RSL in blue, RSO in red, and CC in green). Error bars represent SEM.

Following Over et al. (2007), we first looked at search fixations from all trials and determined mean fixation duration and saccade amplitude as a function of their ordinal number in the trial (see Figure 9). To avoid averaging over small amounts of data we used a similar upper cutting point (5% of trials showing that number of fixations). In our case this corresponded to ordinal numbers 11 for RSL, 6 for RSO and 7 for CC. As can be seen in the figure our data replicates the effect of a longer initial fixation previously reported for visual search tasks (Hooge & Erkelens, 1996; Over et al., 2007; van Loon et al., 2002). Interestingly, the same pattern appeared in the three experiments: fixation durations *decreased* with ordinal fixation number while saccades amplitudes gradually *increased*. This pattern is exactly the *opposite* that would be expected if a coarse-to-fine strategy were used.

**Figure 10.**
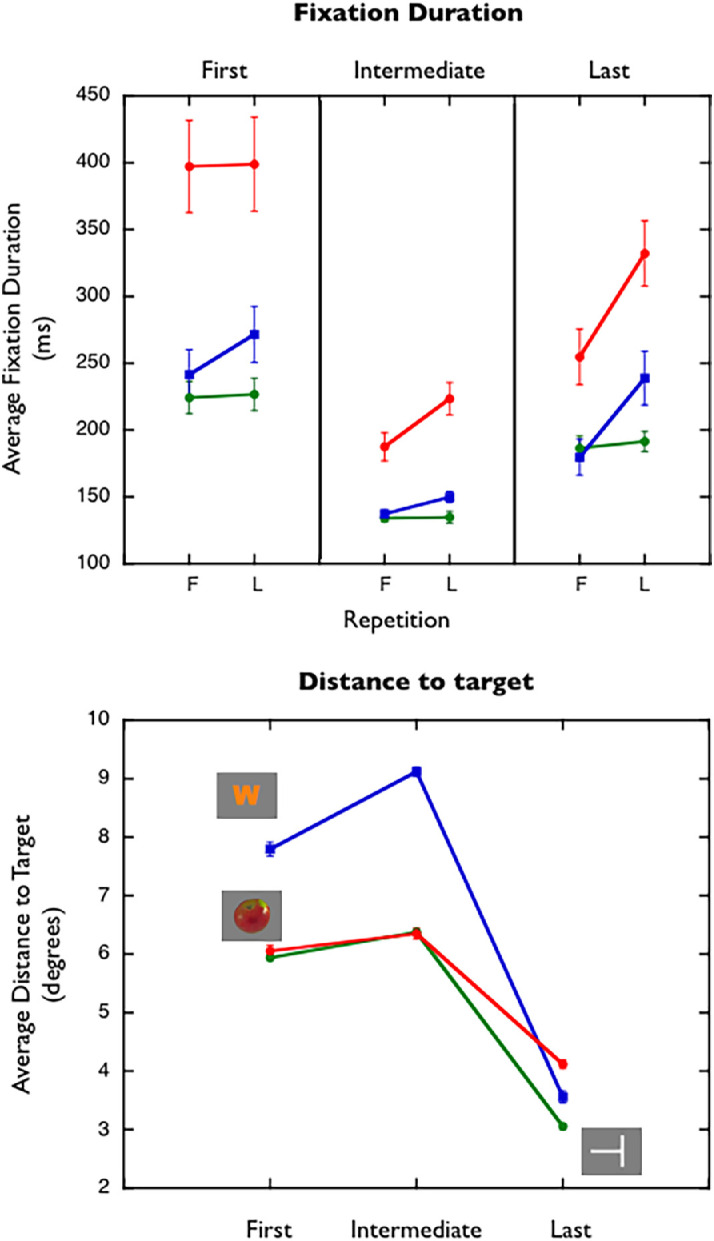
Mean fixation duration (top) and distance to target (bottom) for initial, intermediate and last search fixations performed in the trial. On the top figure only trials from the first (F) and last (L) repetition of the display are plotted. On the bottom figure all trials are included. Each task is represented by a different color (RSL in blue, RSO in red, and CC in green). Error bars represent SEM.

To disentangle possible effects of repetition we also compared fixation durations separating *search* fixations in each trial in three groups: initial fixation, last fixation (usually right before the target is fixated) and remaining, or intermediate, fixations (variable number in each trial). In this case all search fixations from each trial are included (unlike the previous analysis). These results are plotted in the top part of Figure 10, comparing the first and the last repetition of the display in each experiment. In the three experiments the initial search fixation, as well as the last search fixation on that trial had a longer duration. Interestingly, repetition very clearly modulated fixation durations: intermediate and last fixations showed an increase in duration between the first and the last repetition, which was more pronounced in RSL [*intermediate*: *t*(1324) = −2.585, *p* = 0.0098; *last*: *t*(282) = −2.4589, *p* = 0.0145] and RSO [*intermediate*: *t*(726) = −2.1537, *p* = 0.0316; *last*: t(519) = −2.4257, *p* = 0.0156]. We also looked at how far away from the target location were these fixations (bottom part of Figure 10). In the three experiments we found the same pattern: the very last search fixation was, on average, much closer to the target than all the others, usually just 3° or 4° away (see also variations over repetitions in Figure 8). This clearly suggests that the last search fixation last longer because the target has been detected in the periphery.

## Discussion

In this article we report the results of three different experiments that analyzed gaze dynamics in visual search. The three tasks differed in many aspects, but all shared one common aspect: the repetition of targets and search displays. Our results clearly show a reduction in reaction times and number of fixations over repetitions in the three cases. This is coherent with previous studies showing an improvement in search for repeated target items (Höfler, Gilchrist, & Körner, 2014; Körner & Gilchrist, 2007) and for repeated display configurations (Chun, 2000; Chun & Jiang, 1998). However, the most interesting results are those related to eye movement dynamics and what they suggest regarding visual search strategies. We systematically found a difference in duration between *search* and *target* fixations: search fixations were usually short (mostly below 200 msec), while target fixations had much longer durations (averages over 300 msec or higher). These results clearly replicate those of Wu and Kowler (2013). In our case target fixations were always followed by a motor response, which might explain why our durations are even longer than those reported by Wu and Kowler (2013). But, as they suggested, we think that this systematic difference between targets and non-targets also reflects some of the characteristics of the search process.

Similar results have been found in other studies, for example using a pattern discrimination task (Gould & Dill, 1969), a contextual cueing task (Tseng & Li, 2004) or a comparative visual search task (Pomplun et al., 2001). From the point of view of what we know about the role of vision in the control of actions it seems reasonable to find these differences in fixation durations. In complex natural tasks it is usually quite obvious that fixations are made with the purpose of obtaining specific information (Hayhoe, 2009; Hayhoe & Ballard, 2005). For example, in a model copying task participants often fixated the model twice: once to gather information about the next piece color and a second time to determine its location (Ballard, Hayhoe, & Pelz, 1995), and these fixations differed in duration (Hayhoe, Bensinger, & Ballard, 1998). In a block picking task performed in virtual reality different fixations were also used to determine object color and height (Droll & Hayhoe, 2007; Triesch, Ballard, Hayhoe, & Sullivan, 2003). In daily activities, like making a sandwich or a cup of tea, fixations can be classified depending on their *role* for the success of the current action, as locating, directing, guiding and checking fixations (Land & Hayhoe, 2001). Fixation durations in each case also vary, with locating fixations showing much shorter durations than directing or guiding fixations (Hayhoe, Shrivastavah, Mruczek, & Pelz, 2003; Land, Mennie, & Rusted, 1999). All these results suggest that fixations are terminated once the particular information that is required at that point in the task has been acquired.

So how does this translate to visual search? Even when participants seem to be passively looking at a screen, not all fixations have the same role. It is possible to separate exploration from targeting (Wu & Kowler, 2013; Wu & Wolfe, 2022) or detection from verification (Pomplun et al., 2001). In terms of information acquisition, each one of the *search fixations* can be understood as using a binary decision criterion: is the target detected or not. *Target fixations*, on the other hand, most likely reflect the characteristics of the response process. As Kotowicz, Rutishauser, and Koch (2009) have suggested, the function of the final fixation on the target might be primarily to gain confidence before a response is given. Therefore, these fixations have a different role from those performed during search. The target might have been detected at the periphery in the previous fixation and is fixated just for confirmation. This seems coherent with the last search fixation being closer to the target location, as we found (Figure 10). It could also explain why targeting saccades tend to show smaller amplitudes than search saccades, something that occurred in our study and also in the study by Wu and Wolfe (2022).

A second important result that was consistent across the three studies is that repetition of target and search display led to changes in the different oculomotor variables. We found that search fixations systematically *increased* duration over repetitions in the three tasks (Figure 6). Target fixations, however, tended to *decrease* duration. Increases in average fixation durations have been reported in other studies, for example for repeated contexts in a contextual cueing task (Zang, Jia, Müller, & Shi, 2015). The increase in the duration of *search fixations*, together with the general decrease in the number of fixations needed to perform the task over repetitions, cannot be explained as the result of a general coarse-to-fine strategy (Over et al., 2007). In our opinion, this increase reflects a progressive change in search strategies. Participants are probably delaying saccades to locate targets (for example, by considering elements further away in the periphery) or to improve saccadic accuracy, or both. This is coherent with the last search fixation increasing duration over repetitions (Figure 10). Other authors have also reported an increase in fixation durations when greater amounts of information can be extracted from the display (Laubrock, Cajar, & Engbert, 2013). Although saccades to the target might occur by chance, it is more probable that information from the periphery is being used to locate the target (Wu & Wolfe, 2022). Such identification might be initially incomplete, but it can improve over repetitions under reliable conditions, like the ones used in our experiments. For example, in both RSL and RSO the search display was *identical* in all trials, so participants could learn to filter out targets from distractors, even in the periphery (Wu & Kowler, 2013). This would lead to an increase in fixation durations, especially so for the last search fixation, which is what we find. An improvement in saccadic accuracy with repetitions is also coherent with our results: in all tasks saccade amplitudes decrease over repetitions (Figure 7) and search fixations get gradually closer to the target location (Figure 8).

Another factor that could have a role in our experiments is memory. Because target and display locations were mostly stable (only in CC some new displays were presented, intermixed with repeated configurations), participants could learn some of the items’ locations and use memory to guide gaze directly to the target. This has been seen in other, more complex tasks (i.e., Aivar, Hayhoe, Chizk, & Mruczek, 2005; Epelboim et al., 1995; Karn & Hayhoe, 2000). Still, in these three experiments we seldom detected direct saccades to the target. In most cases participants needed to perform *at least* two saccades to reach the target location. Since all our displays were quite crowded it is not surprising that direct guidance to the target location was not possible (Vlaskamp & Hooge, 2006). Nevertheless, the decrease in saccade length that we found in the three tasks, which suggests improved guidance during search, could partially result from knowledge of the display. This could explain the different slopes obtained in each task. RSO provided the best conditions for participants to take advantage of spatial memory, with bigger items and fewer elements. And we found that saccade lengths showed the biggest change over repetitions, both for search and targeting saccades. The smallest effects (although still significant) were found in CC. This makes sense, because in this task only half of the displays were repeated in each block, and there were eight different displays instead of just one. Finding, as we do, that even under these conditions it is possible to see a gradual change in search strategies suggests that these effects result from the combination of multiple factors (i.e., a better target template, increased discrimination of items in the periphery, improved saccadic accuracy).

Some of our results also suggest that search was performed at the optimal level. As different authors have proposed (Epelboim et al., 1995; Steinman, Pizlo, Forofonova, & Epelboim, 2003), participants do not always need to land at the center of a target to solve the task. They can do so from locations that are just close enough to provide the information needed. For RSL and CC, in which items were small, we see that targeting saccades normally land at a distance of less than half a degree from the center of the target. These values do not change much over trials (see bottom part of Figure 8). But in the case of RSO we found, quite surprisingly, that distance to the target in the case of target fixations *increased* over repetitions. In the last block of trials participants could determine that the item was the correct target from a distance of 0.6 degrees from its center. Considering that objects in this task occupied an area of about 1.75°, these fixations are still *inside* the item, but further away from its center. This probably means that, after multiple trials, items do not need to be exactly at the fovea to be recognized. In fact, in RSO we also found that targeting saccades decreased length over repetitions (see bottom part of Figure 7). This suggests that the final saccade to the target was made from a nearer location and just to the general area occupied by the object, which was enough to determine that it was the target.

Regarding fixation durations we also found that *target* fixations tended to decrease duration over repetitions, although this effect was more pronounced in RSL and barely significant in CC. Such decrease in the duration of *target* fixations might reflect an improvement in the decision process over repetitions (Kunar, Flusberg, Horowitz, & Wolfe, 2007; Sewell, Colagiuri, & Livesey, 2018). Other studies have also reported decreases in decision times in repeated search tasks (Solman & Kingstone, 2014). If that were the case, it makes sense that we find different effects depending on the task. Items in RSO were big and very easily discriminable (different shapes and colors), so probably the final fixation on the target, if it was made to confirm target identity (Kotowicz et al., 2009), reached an optimal duration early on, once the objects were known. In terms of search performance RSL was the hardest of the three tasks, so it is not surprising to see the biggest effect on target fixation durations in this case: over repetitions, participants most likely improved their ability to determine letter identities and needed less time at the target to confirm their response.

All these results are very relevant for our understanding of visual search. Current models of visual search (i.e. Guided Search 6.0, see Wolfe, 2021) are based on the idea of the *Functional Visual Field* (FVF): the area around the current fixation point in which visual information can be processed (Sanders, 1970). Different authors have shown that the size of the FVF is linked to search difficulty (Hulleman & Olivers, 2017; Ikeda & Takeuchi, 1975). However, it is generally assumed that fixations have a *constant* duration, with an estimated value of about 250 msec (Hulleman & Olivers, 2017). This estimation is probably based on the average values obtained from all fixations detected in a trial, since not many authors have analyzed those variations that occur intra trial. In our case most search fixations had durations below 200 msec. (see Figure 5), but global averages over all fixations yielded values well above 200 msec. Finding such short fixations during search is not surprising, since other authors have reported similar results (i.e., Findlay, Brown, & Gilchrist, 2001; McPeek, Skavenski, & Nakayama, 2000). Most importantly, our results suggest that the functioning of the FVF is more complex than usually assumed, in agreement with previous criticisms (Laubrock, Engbert, & Cajar, 2017; Shi, Zang, & Geyer, 2017). The modulation of fixation duration that we find in the three tasks, together with other changes in oculomotor performance over repetitions, suggests a dynamic adjustment of the *size* of the FVF. Over trials participants seem to get better at discriminating the targets from the distractors and so can consider more elements in each fixation. This results in a more optimal selection of the next fixation location (as reflected in smaller saccadic amplitudes and a decrease in the distance to the target). This is in agreement with previous results that have shown that the size of the FVF can change during the task or between experimental sessions (Ikeda & Takeuchi, 1975; Motter & Simoni, 2008). There is also previous empirical evidence suggesting a connection between fixation durations and the size of the FVF. For example, when fixation durations are artificially increased, due to the “pip-and-pop” effect (van der Burg, Olivers, Bronkhorst, & Theeuwes, 2008), visual search improves (Zou, Müller, & Shi, 2012). These authors interpret this improvement as the result of increased information uptake. An interesting aspect is that such modulation of fixation duration depends on the availability of visual information: it only occurs when participants can take advantage of foveal or peripheral information. If the visual signal is degraded to the point of providing little useful information, fixation durations revert to very stable values (Laubrock et al., 2013). Since in two of our tasks the display did not change between trials, participants could take maximum advantage of peripheral information, making the investment on longer fixations very beneficial (Laubrock et al., 2013; Wu & Kowler, 2013). Those two tasks showed the biggest effects regarding modulation of search fixation durations.

Overall, our results pinpoint to the need for a more complex and dynamic conceptualization of the FVF. Wu and Wolfe (2022) have already suggested that there are at least three types of FVF: resolution FVF, attentional FVF and exploratory FVF. They have also shown that they can differ in size and characteristics. Our results fit nicely with these distinctions. Search fixations show the inner workings of the exploratory FVF, whereas target fixations are more related to the attentional FVF. What we see is that general experience with the task, and target and display repetition affect them both differently. So even in a simple visual search task presented on a computer screen not all fixations have the same role, as Wu and Kowler (2013) already suggested. The repetition effects that we report also agree with their idea of a strategic adjustment of saccadic timing according to momentary task demands during search. Kowler believed that the role of saccadic planning is to maximize performance. Our results provide details of how that is done in the context of a visual search task.
